# Posterior airway space following mandibular reconstruction using microvascular free flaps in oral cancer patients in relation to radiotherapy: a single-center study

**DOI:** 10.3389/fonc.2026.1875090

**Published:** 2026-08-25

**Authors:** Stefan Heene, Leonard Simon Brandenburg, Klara Klöble, Wiebke Semper-Hogg, Rainer Schmelzeisen, Marc Christian Metzger, Jonas Wüster

**Affiliations:** Department of Oral and Maxillofacial Surgery, University Medical Center Freiburg, Faculty of Medicine, Albert Ludwig University of Freiburg, Freiburg im Breisgau, Baden-Württemberg, Germany

**Keywords:** head and neck cancer, microvascular surgery, oral cancer, oral and Maxillofacial surgery, posterior airway space

## Abstract

**Introduction:**

The posterior airway space (PAS) is of considerable clinical relevance, as airway obstruction in this region may compromise patient well-being, manifesting as symptoms ranging from acute dyspnea to chronic obstructive sleep apnea. Although previous studies have demonstrated significant alterations in the PAS following oral and maxillofacial surgical procedures, changes in the PAS among patients with oral cancer undergoing microvascular reconstruction remain insufficiently investigated. This study aimed to evaluate changes in PAS following mandibular resection and microvascular free flap reconstruction, focusing on the impact of radiotherapy.

**Methods:**

In this retrospective, single-center study, patients with oral cancer treated between January 1, 2020, and January 1, 2025, were assessed. Patients were eligible for inclusion if they had undergone mandibular resection followed by primary microvascular reconstruction and if preoperative and postoperative computed tomography (CT) scans with a maximum slice thickness of 1 mm were available. Imaging data were segmented using 3D Slicer (The Slicer Community, www.slicer.org), and PAS volume was quantified in cubic centimeters (cm³). Intraindividual changes in PAS volume were subsequently analyzed, and potential influencing factors, such as adjuvant radiotherapy, defect type, and reconstruction method, were evaluated.

**Results:**

A total of 42 patients (18 females, 24 males; mean age 66 ± 13 years) were included in the analysis. Overall, PAS volume increased by a mean of 11.6% (95% CI: −6.8% to 29.9%) on the first postoperative CT scan. At one-year follow-up (mean 381 days), PAS volume had increased significantly by 58.5% (95% CI: 39.7% to 77.2%) compared with baseline (p = 0.0023). The increase in PAS volume was significantly greater in patients who received adjuvant radiotherapy (p = 0.0266). Furthermore, multiple linear regression identified neck dissection, defect type, reconstruction method, and adjuvant chemotherapy as independent factors significantly associated with changes in PAS volume at the one-year follow-up.

**Discussion:**

Mandibular resection followed by microvascular reconstruction is associated with a significant overall increase in PAS volume in patients with oral cancer. However, multiple factors - particularly adjuvant radiotherapy – are significantly associated with the extent of PAS alteration.

## Introduction

Cancers of the oral cavity represent a major global health burden and account for a substantial proportion of head and neck malignancies. Recent global estimates report more than 370,000 new cases annually, with oral squamous cell carcinoma being the predominant histological subtype (1). Surgical resection remains the primary curative treatment modality for most patients with oral cancer. In locally advanced disease, surgical therapy frequently requires partial or segmental resection of the mandible together with adjacent soft tissues. To restore mandibular continuity and preserve oral function, such defects are routinely reconstructed using microvascular free tissue transfer (2–4).

Beyond functional and aesthetic outcomes, mandibular resection and reconstruction may alter upper airway morphology. The posterior airway space (PAS) defines the anteroposterior dimension of the pharyngeal airway from the palate to the epiglottis, bounded anteriorly by the velum and posteriorly by the pharyngeal wall (5, 6). Alterations in PAS dimensions are clinically relevant, as airway narrowing may lead to intermittent or persistent obstruction. Reduced airway dimensions have been associated with obstructive sleep apnea and related systemic complications, including hypertension, arrhythmias, myocardial ischemia, heart failure, and stroke (7). Craniofacial skeletal morphology is a key determinant of PAS dimensions. Consequently, the relationship between mandibular position and airway geometry has been extensively studied in orthognathic surgery (5, 8, 9). In particular, posterior displacement of the mandible has been shown to reduce airway dimensions and may predispose patients to airway obstruction (10). However, although mandibular resection and reconstruction in oncologic surgery can lead to substantial alterations of mandibular position and surrounding soft tissues, the potential impact of these procedures on PAS morphology has received comparatively little attention.

Previous studies have demonstrated a high prevalence of sleep-disordered breathing and OSA among patients treated for oral and oropharyngeal malignancies (11). Changes in upper airway function have also been reported following extensive soft-tissue resection and free flap reconstruction (12, 13). Moreover, historical observations indicate that loss of mandibular continuity, particularly after anterior mandibulectomy without adequate reconstruction, may contribute to postoperative airway obstruction and sleep apnea (14). Nevertheless, evidence specifically relating bony mandibular reconstruction to postoperative airway morphology and functional sleep-related breathing outcomes remains limited.

Moreover, several factors inherent to oncologic reconstruction - including the extent and location of mandibular defects, reconstructive techniques, neck dissection and adjuvant treatments such as radiotherapy - may further influence postoperative airway morphology. Understanding these effects may be clinically relevant, as certain reconstructive configurations or treatment modalities could predispose patients to unfavorable airway changes.

Virtual surgical planning has improved the accuracy and efficiency of mandibular reconstruction by facilitating precise restoration of mandibular continuity, projection, symmetry, and facial contour (15). In addition to optimizing skeletal reconstruction, accurate three-dimensional positioning of the reconstructed mandible may help preserve the attachment and spatial orientation of the tongue, suprahyoid musculature, and floor-of-mouth tissues that contribute to upper airway stability (16). However, whether virtual surgical planning and improved anatomical reconstruction translate into measurable benefits for postoperative PAS morphology or functional airway outcomes has not yet been sufficiently investigated.

Furthermore, conventional CT-based volumetry provides a static representation of upper airway anatomy. Dynamic CT and cine-MRI studies have shown that airway dimensions and shape vary throughout the respiratory cycle and during sleep (17, 18). Static total airway volume therefore does not capture tissue compliance, respiratory phase-dependent changes, or dynamic airway collapsibility and cannot be regarded as a direct surrogate for functional airway patency or the presence of OSA. Nevertheless, longitudinal CT-based volumetry allows objective assessment of postoperative anatomical changes and may help identify surgical and treatment-related factors associated with alterations in airway morphology.

This study aimed to assess changes in posterior airway space (PAS) volume following mandibular resection and microvascular reconstruction in oral cancer patients, and to identify factors influencing postoperative airway alterations, including defect type, reconstructive approach, and adjuvant radiotherapy.

## Methods

### Ethical approval

The study has been approved by the Ethics Committee of the University of Freiburg (No. 30/20) and was registered in the German Clinical Trials register (No. DRKS00029159). The study was performed in accordance with the Helsinki Declaration of 1964 as revised in 2013 (19).

### Patient consent statement

In accordance with the statement of the ethics committee, individual consent of the patients was not necessary as the data was processed retrospectively after anonymization.

### Inclusion and exclusion criteria

For this retrospective study, patients who underwent segmental resection of the mandible due to oral cancer and received reconstruction with a microvascular free flap between January 1, 2020, and January 1, 2025, at the Department of Oral and Maxillofacial Surgery of the Medical Center of the University of Freiburg, Freiburg im Breisgau, Germany, were assessed. Patients were eligible for inclusion if computer tomography (CT) scans with a maximum slice thickness of 1 mm were available preoperatively and within the first six postoperative months or approximately one-year post-surgery. CT scans of intubated patients were excluded.

### Data acquisition

All CT examinations were acquired according to the institutional radiological protocol using the same multidetector CT scanner (SOMATOM Definition Flash; Siemens, Erlangen, Germany). Owing to the retrospective study design, standardization of head posture, tongue position, breathing phase, and swallowing during image acquisition was not feasible. However, only examinations with sufficient image quality and comparable head positioning were included for analysis.

Medical records were reviewed retrospectively, using electronic patient charts. This research was done without patient involvement. Moreover, patients were not invited to comment on the study design and were not consulted to develop patient-relevant outcomes or interpret the results. Additionally, to the required Digital Imaging and Communications in Medicine (DICOM) data, we identified several patient-specific factors to test the influence of potential confounding factors (see below). The collected data was arranged using a Microsoft Excel spreadsheet (Microsoft Excel^®^, Version 16.0, Microsoft Corporation, NM, USA).

### Primary results

We hypothesized that mandibular resection and reconstruction influence the PAS, depending on the T stadium, defect type, type of reconstruction, and the presence of other factors like neck dissection and adjuvant radio- and chemotherapy. Therefore, the PAS was measured preoperatively (t_0_) and compared to distinct postoperative measurements: The first postoperative CT scan was assigned to t_1_ if it was obtained less than 180 days post-surgery. CT scans obtained later than 180 days post-surgery were assigned to t_2_; if several CT scans obtained later than 180 days post-surgery were available, the one closest to 365 days post-surgery was included. Afterwards, measurements were analyzed in the overall study collective and within certain confounders.

**Figure 1:**
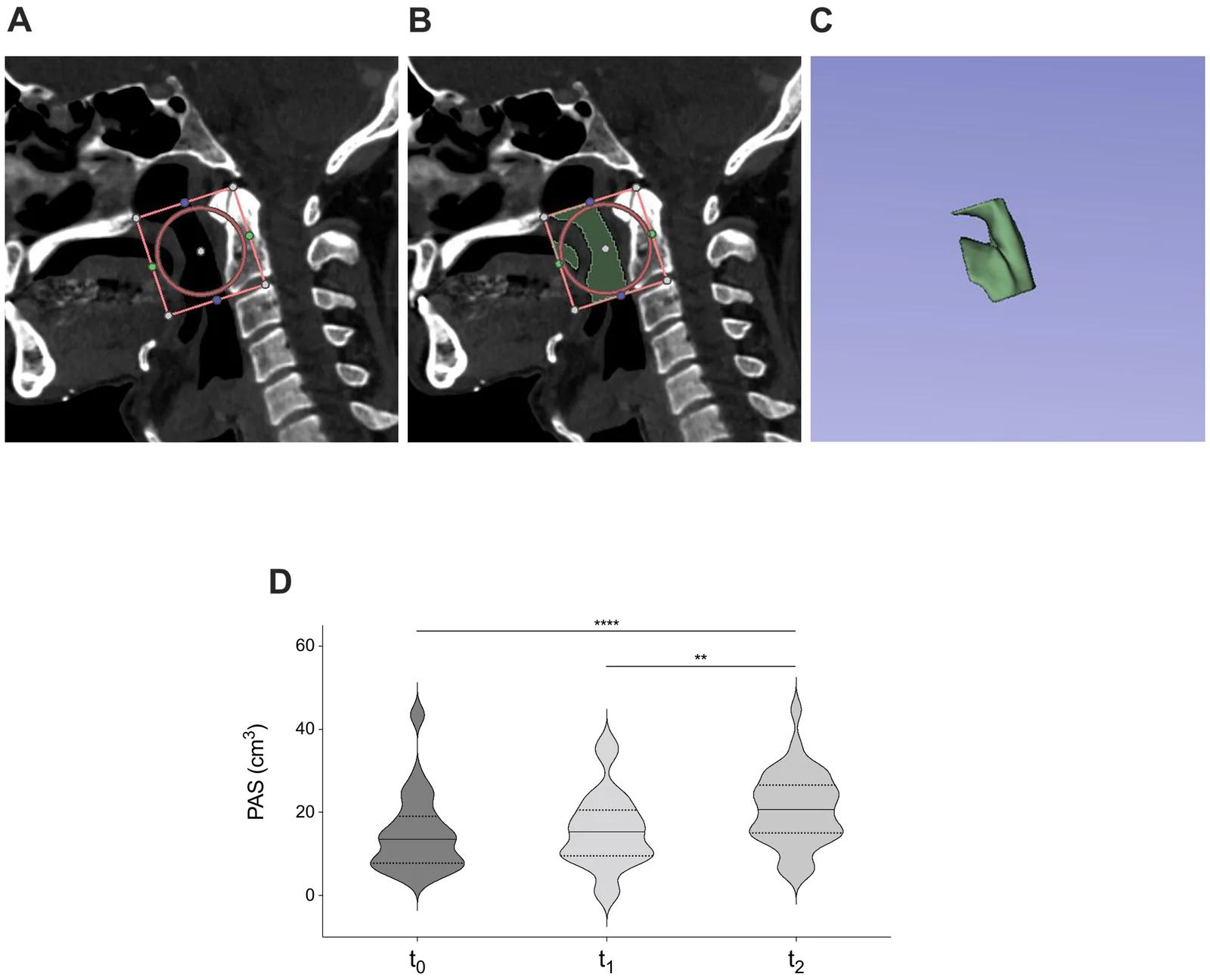
Defining and measuring the PAS. **(A)** Exemplary definition of the ROI to measure the PAS in a sagittal view CT scan in 3D Slicer. **(B)** Exemplary segmentation of the air inside the ROI to measure the PAS in a sagittal view CT scan in 3D Slicer. **(C)** Exemplary three-dimensional rendering of the segmented PAS in 3D Slicer. **(D)** Violin plot of absolute PAS volumes through all three timepoints; **p < 0.01, ****p < 0.0001.

To measure the PAS, DICOM datasets were imported into 3D Slicer (version 5.8.1, The Slicer Community, www.slicer.org (20, 21)). The region of interest (ROI) was defined as a cuboid-shaped volume using the Volume Rendering module. For each CT dataset, the ROI was individually reoriented according to the palatal plane, which served as the cranial boundary. The caudal boundary consisted of a plane parallel to the palatal plane passing through the superior margin of the epiglottis. The anterior boundary was defined by a plane perpendicular to the cranial and caudal planes passing through the posterior nasal spine (PNS), whereas the posterior boundary was formed by the posterior pharyngeal wall. Airway segmentation was performed using the Segment Editor module employing threshold-based segmentation (−1024 to −300 Hounsfield units), followed by manual refinement to remove segmentation artefacts and disconnected air-containing structures. No additional image filtering or smoothing was applied. After segmentation, the software automatically calculated the enclosed airway volume in cubic centimeters (cm³) (**Figures 1A–C**).

### Confounders

Mandibular defects were classified according to Jewer and Boyd (22). Reconstructions comprised free fibula flaps (FFF), deep circumflex iliac artery (DCIA) flaps, scapula free flaps (SFF), radialis flaps and rectus abdominis flaps. Further considered potential confounders were T stadium, sex and age, neck dissection, and adjuvant radio- and chemotherapy.

### Statistics

Group differences were assessed using unpaired two-sample t-tests, one-way ANOVAs, as well as mixed-effects analyses followed by Tukey-Kramer *post hoc* tests for group × timepoint comparisons. Multiple linear regression was used to combine all potential confounders to a single model. Normality was confirmed prior to all parametric tests using the Shapiro-Wilk test. A p-value < 0.05 was considered statistically significant. All statistical analyses and visualizations were performed in GraphPad Prism (version 9.0; GraphPad Software, San Diego, CA, USA).

## Results

### Demographic and clinical characteristics of the study population

A total of 42 patients (18 females, 24 males; mean age 66 ± 13 years) were included in this study. Among these, 33 patients had imaging within the first 180 days after surgery, and 35 patients had at least one CT examination at a later time point. In 26 cases, imaging was available for both investigated time points. The preoperative CT examination (t_0_) was performed on average 26 ± 13 days before surgery, while the first postoperative scan (t_1_) was obtained after a mean of 58 ± 53 days. The second postoperative CT examination included in the analysis (t_2_) was conducted after an average of 381 ± 169 days. Two CT scans from the < 180-day group were excluded due to patient intubation.

Surgery was performed for basal cell carcinoma, sarcoma, and metastasis of breast carcinoma in one patient each. In the remaining 39 patients, the underlying diagnosis was squamous cell carcinoma.

The most common defect pattern (n = 26) comprised class L defects (according to Jewer and Boyd), followed by 11 class LCL defects. Reconstruction was most frequently performed using a FFF (n = 28), followed by DCIA flaps (n = 6) and SFF (n = 4). Adjuvant radiotherapy was administered in 17 patients. Additional subgroup sizes are summarized in **Table 1**.

**Table 1:** Demographic and clinical characteristics of the study population and subgroups.

Age (years)	66 ± 13
Sex (n)	
- Male - Female	24 18
Disease (n)	
- OSCC - BCC - Sarcoma - Metastasis	39 1 1 1
T stadium (n)	
- 4a - 3 - 2 - 1	25 9 3 3
Type of reconstruction (n)	
- Fibula - DCIA - Scapula - Radialis - Rectus abd.	28 6 4 3 1
Defect type (n)	
- L - LCL - H - C	26 11 4 1
Adjuvant radiotherapy (n)	
- Yes - No	17 25

### Absolute changes in PAS volumes

Analysis of the absolute measurements across all time points revealed that the PAS increased substantially in volume following surgery. Within the first postoperative CT scan, values increased from 14.86 ± 9.15 cm³ (t_0_) to 15.60 ± 9.00 cm³ (t_1_), representing an approximate 11.6% (95% CI: −6.8% to 29.9%) increase relative to preoperative measurements. By the one-year follow-up, a further significant increase in volume to a mean of 20.81 ± 8.74 cm (3) was observed (p = 0.0023), resulting in an average PAS volume that was 58.5% (95% CI: 39.7% to 77.2%) larger than preoperatively (p < 0.0001; Hedges’ *g* = 0.69; **Figure 1D**).

### Subgroup-specific, relative changes in PAS volume

**Figure 2:**
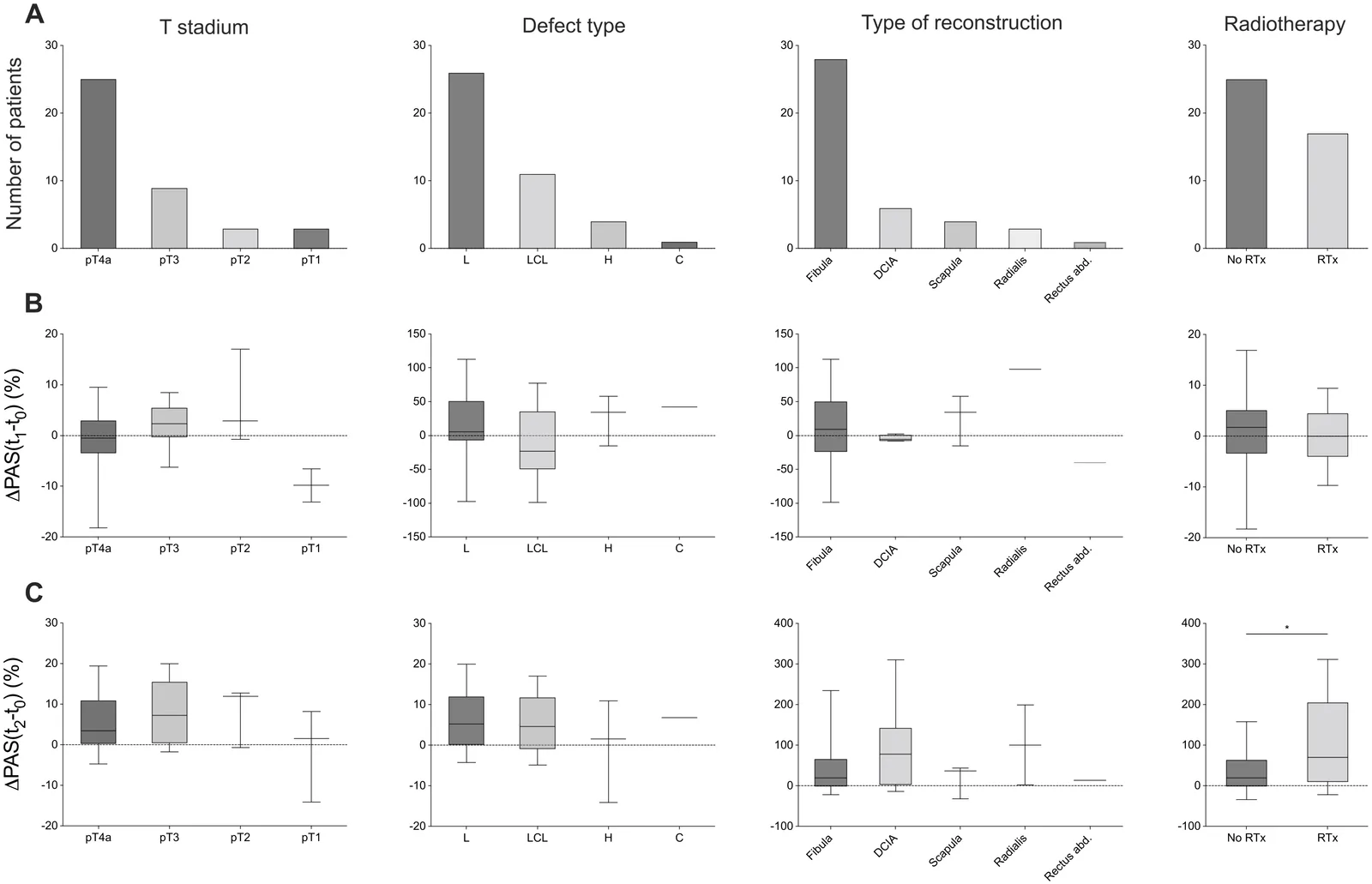
Subgroup-specific relative changes in PAS. **(A)** Number of patients depending on T stadium, defect type, type of reconstruction and radiotherapy. **(B)** Relative changes in PAS volume in the first postoperative CT scans compared to the preoperative measurements ΔPAS(t_1_-t_0_) depending on T stadium, defect type, type of reconstruction and radiotherapy. **(C)** Relative changes in PAS volume in the one-year follow-up CT scans compared to the preoperative measurements ΔPAS(t_2_-t_0_) depending on T stadium, defect type, type of reconstruction and radiotherapy; *p < 0.05.

However, when relative changes in PAS volume were analyzed across subgroups, a less uniform pattern emerged. In several patient groups, the PAS volume actually decreased on average in the first postoperative imaging. This included patients with tumors at the pT4a stage, large midline-crossing defects (LCL), and reconstructions performed using DCIA flaps (**Figure 2B**). Nevertheless, even in these patient groups, a marked increase in PAS volume was observed at the one-year follow-up compared with the preoperative imaging (**Figure 2C**).

The only statistically significant difference in subgroup-specific relative PAS changes was observed at the one-year follow-up between irradiated and non-irradiated patients. While the PAS increased by approximately 34.7% (95% CI: 12.2%–57.2%) in non-irradiated patients after one-year, irradiated patients showed a mean volume increase of 98.1% (95% CI: 36.9%–159.4%) (p = 0.0266; Hedges’ *g* = 0.80; **Figure 2D**).

### Regression analysis for trends in relative changes in PAS volume

To evaluate the effects of all collected variables on relative PAS change, multiple linear regression analyses were performed. In the first postoperative assessment, only the T stage showed a significant influence on PAS change in this model.

At the one-year follow-up, however, defect class, the type of reconstructive graft used, the performance of a neck dissection, and the administration of adjuvant therapy all had a significant influence on the relative change in PAS (**Table 2**).

**Table 2:** Multiple linear regression of all variables (t_2_-t_0_).

Variable	Estimate	Standard error	95% CI (asymptotic)	|t|	P value	Significance
Sex (male)	21,67	31,87	-45,90 to 89,23	0,6797	0,5064	ns
Age	0,04321	1,418	-2,964 to 3,050	0,03046	0,9761	ns
Flap type (SFF)	78,61	36,46	1,317 to 155,9	2,156	0,0466	*
Flap type (Radialis)	43,78	73,99	-113,1 to 200,6	0,5917	0,5623	ns
Flap type (DCIA)	202,7	79,96	33,19 to 372,2	2,535	0,0221	*
Flap type (Rectus abd.)	-165,2	79,26	-333,3 to 2,784	2,085	0,0535	ns
ND (no)	208,1	69,01	61,86 to 354,4	3,016	0,0082	**
RTx (yes)	19,63	30	-43,97 to 83,23	0,6543	0,5222	ns
Chemo (yes)	90,42	38,79	8,181 to 172,7	2,331	0,0332	*
Jewer-Boyd (C)	153	83	-22,91 to 329,0	1,844	0,0838	ns
Jewer-Boyd (LCL)	91,44	31,82	24,00 to 158,9	2,874	0,011	*
Jewer-Boyd (H)	215,3	116	-30,65 to 461,2	1,856	0,082	ns
pT4a	-21,44	31,55	-88,32 to 45,44	0,6795	0,5065	ns
pT1	-152,9	82,6	-328,0 to 22,17	1,851	0,0826	ns
pT2	-14,8	54,42	-130,2 to 100,6	0,2719	0,7891	ns

* p < 0.05; ** p < 0.01; ns, not significant.

## Discussion

The aim of this study was to analyze the influence of segmental mandibulectomy and primary microsurgical mandibular reconstruction on the posterior airway space (PAS) in patients with oral cancer. Furthermore, we sought to evaluate potential factors influencing changes in PAS volume, including tumor stage, defect type, choice of reconstructive transplant, and adjuvant radiotherapy. In line with previous studies, our results demonstrated an overall significant increase in PAS volumes following mandibular resection and reconstruction (23, 24). However, specific subgroup characteristics were associated with a relative decrease in PAS volumes.

In particular, large midline-crossing defects (LCL) were associated with a relative PAS decrease within the first postoperative imaging. A possible explanation may be a slight posterior positioning of the reconstructed mandible, intentionally performed to achieve functional occlusion, neomandibular stability, and facilitate subsequent dental rehabilitation, in line with current surgical recommendations (25, 26). Such positioning may partially reduce the anterior airway space and thereby limit PAS expansion.

With regard to the type of reconstructive transplant, it is noteworthy that particularly FFF and DCIA flaps showed more pronounced PAS alterations compared with other graft types. This finding is of special clinical relevance, as the FFF is widely regarded as the gold standard for complex and especially multi-segment mandibular reconstructions. Given its frequent use in extensive defects, a detailed understanding of its impact on postoperative airway morphology is essential. The observed changes in PAS may be related to the specific structural and vascular characteristics of these grafts. The FFF, for example, consists predominantly of dense cortical bone with limited cancellous components, which may influence its remodeling capacity and adaptation under functional load. Similarly, DCIA flaps provide substantial bone volume but exhibit distinct perfusion patterns and remodeling dynamics. In the context of large and complex defects - where these flaps are most commonly applied - both the extent of reconstruction and the biomechanical properties of the graft may contribute to alterations in mandibular positioning and surrounding soft tissue tension, thereby affecting PAS. These findings underline the importance of considering transplant-specific effects when evaluating postoperative airway changes, particularly in cases where FFF is used as the reconstructive standard.

Radiotherapy represents another important factor, as it can induce dose-dependent tissue damage (27). Tumor stage and the extent of resection must be considered in this context, as advanced tumor stages (T3/T4) and larger defects are typically associated with an indication for adjuvant radiotherapy according to current oncologic treatment guidelines. Consequently, patients undergoing more extensive resections are more likely to receive postoperative irradiation, which may act as a confounding factor when interpreting PAS changes. This becomes evident, for example, in the impaired feasibility and outcomes of secondary microvascular reconstruction in previously irradiated regions, as well as in compromised osseous healing of transplanted bone and reduced survival of dental implants in head and neck cancer patients (25, 28). These effects may also influence postoperative airway morphology, although their specific impact on PAS remains insufficiently understood. In the present study, radiotherapy was therefore considered as a potential influencing factor; however, a more detailed analysis of dose–response relationships and radiation fields was beyond the scope of this work. It is well known that irradiation leads to unfavorable tissue conditions that may complicate microvascular reconstruction (29, 30). In addition, direct effects of irradiation on the upper airway include airway fibrosis, which can lead to PAS volume reduction and airway obstruction (31, 32). Interestingly, patients who received adjuvant radiotherapy in our cohort demonstrated a significantly greater relative increase in PAS volume than non-irradiated patients. At first glance, this finding appears counterintuitive, as radiation-induced fibrosis is generally associated with tissue stiffening and airway narrowing. One possible explanation is that radiation-induced atrophy of the suprahyoid, floor-of-mouth, and pharyngeal musculature, as well as of the transplanted tissue itself, may have exceeded the airway-narrowing effects of fibrosis, thereby reducing the surrounding soft tissue bulk and resulting in a relative increase in measurable airway volume (33–35). However, this proposed mechanism remains speculative and cannot be confirmed by the present study, as MRI-based soft tissue assessment, quantitative muscle volumetry, fibrosis grading, radiation dosimetry, and objective evaluation of radiation-induced tissue changes were not available. Future studies combining volumetric imaging with quantitative assessment of soft tissue alterations are therefore warranted to further elucidate the mechanisms underlying these observations. Furthermore, detailed radiotherapy-related parameters, including radiation dose, treatment technique, radiation field, and the interval between completion of radiotherapy and follow-up imaging, were not consistently available and therefore could not be incorporated into the present analyses.

Taken together, these findings suggest that although mandibular reconstruction generally increases PAS volume, specific clinical factors may modulate postoperative airway morphology and potentially predispose certain patients to PAS reduction during the subacute postoperative phase.

No distinction was made between different reconstructive approaches when using microvascular osseous free flaps. CAD/CAM-assisted techniques have been shown to offer advantages over conventional methods, particularly with regard to the accuracy of the reconstructive procedure. However, previous work has demonstrated that, depending on the flap type used, postoperative deviations from the preoperative plan may still occur (36–38). This may influence the reconstructive outcome and, depending on the defect type, also the posterior airway space (PAS). The accuracy of reconstruction was not specifically assessed in the present study.

Nevertheless, several limitations of this study must be considered. First, the overall study population comprised only 42 patients. Owing to the relatively limited sample size, particularly within individual reconstructive subgroups, the present analyses should be interpreted with caution. Small subgroup sizes may reduce statistical power and increase the risk of type II error. Furthermore, although only clinically relevant variables were investigated, the possibility of overfitting cannot be entirely excluded. Consequently, the multivariable regression analyses should primarily be regarded as exploratory and hypothesis-generating. Larger multicenter studies are warranted to validate these findings. Furthermore, the region of interest (ROI) was manually defined by a single experienced observer following a standardized segmentation protocol. Although this approach ensured methodological consistency throughout the study, formal intraobserver repeatability analyses, interobserver agreement assessments (e.g., intraclass correlation coefficients), and repeated measurements were not performed. Consequently, minor inaccuracies in PAS delineation due to observer-dependent variability cannot be excluded and should be considered when interpreting the volumetric measurements. In addition, the retrospective single-center design may have introduced selection bias and limits the generalizability of our findings. The requirement for both preoperative and postoperative high-resolution CT examinations may have preferentially selected patients undergoing closer radiological surveillance, whereas patients with uncomplicated postoperative courses may have been underrepresented. Moreover, owing to the retrospective nature of the study, complete standardization of patient positioning during CT acquisition was not feasible. Consequently, residual variability related to head and neck position as well as physiological airway dynamics cannot be excluded. As head and neck position is known to influence upper airway dimensions, these factors may have affected the measured PAS volumes independently of the surgical intervention and should therefore be considered when interpreting the findings (39–41). Moreover, we assessed only total PAS volumes. However, parameters such as minimal cross-sectional area may play a crucial role in airway obstruction (42–44). Therefore, further analyses of additional airway characteristics would improve the assessment of airway functionality. Most importantly, this study does not provide data on clinical symptoms or patient-reported outcomes. Although statistically significant postoperative changes in PAS were observed, their direct clinical relevance remains uncertain. Airway volume alone does not necessarily predict respiratory symptoms or sleep-disordered breathing. Furthermore, swallowing function, speech outcomes, and quality-of-life measures were not assessed. Future prospective studies incorporating polysomnography, pulmonary function testing, swallowing assessments, and validated patient-reported outcome measures are required to determine the clinical implications of these anatomical changes.

Despite these limitations, this study contributes to the existing literature by providing valuable insights into factors influencing PAS alterations following mandibular resection and reconstruction in patients with oral cancer.

## Conclusion

Mandibular resection followed by microvascular reconstruction is associated with a significant overall increase in PAS volume in patients with oral cancer. However, several factors, including defect type, reconstruction method, and adjuvant radiotherapy, were significantly associated with the magnitude of PAS alteration. Consequently, certain patient groups may remain at risk for postoperative airway obstruction, which should be considered in clinical practice.

## Data Availability

Requests to access the datasets should be directed to Stefan.heene@uniklinik-freiburg.de.
